# Editorial: Orofacial Pain of Muscular Origin—From Pathophysiology to Treatment

**DOI:** 10.3389/froh.2021.825490

**Published:** 2022-01-14

**Authors:** Nikolaos Christidis, Essam Ahmed Al-Moraissi

**Affiliations:** ^1^Division of Oral Diagnostics and Rehabilitation, Department of Dental Medicine, Karolinska Institutet, Huddinge, Sweden; ^2^Scandinavian Center for Orofacial Neurosciences, Huddinge, Sweden; ^3^Department of Oral and Maxillofacial Surgery, Faculty of Dentistry, Thamar University, Dhamar, Yemen

**Keywords:** muscle pain, human, temporomandibular disorders—TMD, orofacial pain mechanisms, children & adolescents

Although orofacial pain is a relatively new discipline in dentistry, its management has been part of the dental profession since the beginning of the 19th century. The orofacial region is one of the most frequent locations for chronic pain conditions. More recent studies indicate a prevalence of 7–11% [1]. Several studies have reported that orofacial pain is responsible for more than half of health consultations and up to 80% of dental appointments among adolescents [2]. Furthermore, it has also been reported that clinicians (both from medicine and dentistry) feel incompetent handling these patients. Hence, lack of knowledge of orofacial pain is a great barrier to appropriate pain management [3]. Temporomandibular disorders (TMD) is a collective term embracing chronic pain in conditions in the orofacial region affecting the temporomandibular joints (TMJ) or the masticatory muscles (myalgia), and their associated structures [4]. TMDs have a prevalence of approximately 10–20% and are 1.5–2 times more prevalent in women. The most common TMD diagnosis is myalgia. Both myalgia and arthralgia (i.e., pain in temporomandibular joints) are associated with restricted mouth opening capacity, pain in chewing, muscle and joint soreness, headache, and impaired chewing ability [1].

The precise relationship between pain and jaw function is still unknown. There are different theories trying to explain their relationship; however, several studies have failed to prove the fundamentals of these theories. There is still limited knowledge of sex, age, and/or tissue differences in the neurophysiology of healthy human orofacial muscles, and of the pathophysiology of TMD pain of muscular origin [5]. Thus, diagnosis of TMDs is not mechanism-based but relies on certain combinations of symptoms and signs [1].

With this in mind, this Research Topic aimed to increase the knowledge of a common and bothersome condition within the field of oral health, provide new insights into the etiology/pathophysiology of muscular pain, and establish grounds for improved and novel treatment approaches.

This Research Topic holds three published manuscripts, purely original research, involving 12 different authors spread around the globe. Shimada et al. investigated the interaction between glutamate-induced masseter muscle pain and allodynia induced by nerve growth factor (NGF) on pain perception and jaw function in healthy individuals, and any possible sex differences in response. The outcome of the study showed that the spontaneous pain intensity was significantly higher after glutamate than after NGF. Further, the study showed that mechanical sensitization, temporal summation pain and functional measures were all significantly reduced 3 days after NGF injection in both men and women, without sex differences. However, both chewing pain and muscle exertion increased after glutamate injection to a significantly higher degree in women than in men. This suggest that patient reported sex differences in patients with myalgia in the orofacial region may be greater for measures of perceived jaw function. Hence, this could be considered during clinical evaluation/examination, since pain that is worsened by jaw function is one of the key symptoms of orofacial myalgia.

Louca Jounger et al. aimed to investigate if single nucleotide polymorphisms (SNPs) related to monoaminergic neurotransmission (in particular the serotonergic pathway) contribute to pain perception in patients with temporomandibular disorder (TMD) myalgia and if there is a correlation between SNPs and jaw function as well as psychosocial factors such as stress, anxiety and depression. Based on venous blood or saliva samples from 117 individuals, this study showed that participants carrying at least one copy of the rare alleles of HTR2A (rs9316233) and HTR3A (rs1062613) had significantly higher characteristic pain intensity compared with the participants with the homozygous common genotype. Furthermore, the correlation analyses showed several significant positive correlations between characteristic pain intensity, on one hand, and self-reported psychosocial distress and jaw function, on the other hand, for several genotypes, although most of the correlations were weak or moderate. These findings suggest that the polymorphism rs1062613 in the HTR3A gene contributes to pain intensity in patients with orofacial myalgia. This, together with positive interactions between pain variables and psychological factors in genotypes, strengthens the theory that pain and psychological distress are related.

Finally, Al-Khotani et al. aimed to evaluate the association between self-reported pain in the orofacial region (TMD-P) with depression, anxiety, and somatic problems in 466 children and adolescents in Saudi Arabia. The outcome of this study showed that self-reported TMD-P in the children and adolescents was significantly associated with anxiety, depression, somatic symptoms, and social problems, and that the frequencies of anxiety, depression, and somatic disorders were significantly more evident among children and adolescents who suffered from TMD-P. The odds of reporting TMD-P in children and adolescents was 1.4 times for borderline and clinical diagnosis scores for anxiety and withdrawal depression domains, and 2.6 times for the domains of somatic symptoms. Moreover, self-reported TMD-P was twice as prevalent among girls compared to boys. Based on the findings from this study, especially the significant association between psychosocial burden and the presence of self-reported TMD-P, there is a need of an early approach and recognition of children and adolescents with anxiety, depression, somatic symptoms, in combination to problems in the orofacial region.

Taken together, this Research Topic highlights the importance of early recognition and examination of orofacial pain conditions in both children/adolescents and adults, not just to decrease pain but also to reduce the risk of psychosocial implications resulting in decreased quality of life. It also highlights that there could be genetic factors that affects both the presence and intensity of pain as well as psychological distress, and strengthens the theory that these conditions are closely related. Finally, pain with jaw functioning and decreased jaw functioning seem to be important aspects when examining patients with muscular pain in the orofacial region.

## Author Contributions

NC compiled the material and wrote the text. EA-M compiled and edited the text. Both authors contributed to the article and approved the submitted version.

## Conflict of Interest

The authors declare that the research was conducted in the absence of any commercial or financial relationships that could be construed as a potential conflict of interest.

## Publisher's Note

All claims expressed in this article are solely those of the authors and do not necessarily represent those of their affiliated organizations, or those of the publisher, the editors and the reviewers. Any product that may be evaluated in this article, or claim that may be made by its manufacturer, is not guaranteed or endorsed by the publisher.
